# Genome-wide identification and characterization of long noncoding and circular RNAs in germline stem cells

**DOI:** 10.1038/s41597-019-0014-9

**Published:** 2019-03-27

**Authors:** Xiaoyong Li, Geng G. Tian, Yongqiang Zhao, Ji Wu

**Affiliations:** 1 Renji Hospital, Key Laboratory for the Genetics of Developmental & Neuropsychiatric Disorders (Ministry of Education), Bio-X Institutes, School of Medicine, Shanghai Jiao Tong University, Shanghai, 200240 China; 2 Key Laboratory of Fertility Preservation and Maintenance of Ministry of Education, Ningxia Medical University, Yinchuan, 750004 China; 3 Shanghai Key Laboratory of Reproductive Medicine, Shanghai, 200025 China

**Keywords:** Cell biology, Stem cells

## Abstract

Germline stem cells are germ cells at an early developmental stage, so their development is key to ensuring human reproduction. There is increasing evidence that long noncoding RNA (lncRNA) and circular RNA (circRNA) play important roles in the development of germ cells. This data descriptor provides unique lncRNA and circRNA transcriptomic information for mouse germline stem cells. Using the Illumina HiSeqx 2000 system, a total of 511,836,732 raw reads were generated. High-quality transcripts, lncRNAs, and circRNAs were identificated and quantified using the reads, and more precise annotations of lncRNAs (especially 9357 novel lncRNAs) and circRNAs were performed in the germline stem cells. We then analyzed the transcript structures, genetic variants, and the interaction between circRNA and microRNA to provide the basis for subsequent functional experiments. This comprehensive dataset will help advance data sharing and deepen our understanding of mouse germline stem cells, providing a theoretical foundation for research on germ cell development and human reproduction, among others.

## Abstract (summary)

**Table:** 

Design Type(s)	transcription profiling design • cell type comparison design
Measurement Type(s)	transcription profiling assay
Technology Type(s)	RNA sequencing
Factor Type(s)	technical replicate
Sample Characteristic(s)	Mus musculus • testis • ovary

Machine-accessible metadata file describing the reported data (ISA-Tab format)

## Background & Summary

There is growing recognition that cells, especially in mammals, produce many thousands of noncoding transcripts. long noncoding RNA (lncRNA), a group of noncoding RNAs that are longer than 200 bp, are emerging as potent regulators of gene expression, and have dramatically altered our understanding of cell biology under pathological conditions^1,2^. Owing to the strong time- and tissue-specificity of lncRNA expression, there is great potential for novel lncRNA prediction. Circular RNAs (circRNAs) are a recently discovered group of noncoding RNAs identified by the presence of a special circular structure formed by covalent bonds^3,4^. In eukaryotic organisms, circRNAs are mostly present in the cytoplasm, but a few intron-cyclized circRNAs are localized in the nucleus^5^. They are widespread in mammalian cells, sometimes being more common than linear RNA. CircRNAs are also highly conserved and are not easily degraded by RNase. As millions of transcripts are generated by next-generation sequencing, many lncRNAs and circRNAs have been identified, but few have been functionally characterized. In this context, it is important to be able to determine the connections between lncRNAs/circRNAs and their target mRNAs, and to clarify their potential functions. RNA-seq is an effective technology that utilizes the features of next-generation sequencing to study lncRNA and circRNA^6^. It can be used in combination with reference annotation-based assembly, which facilitates the detection of novel isoforms being applied in fundamental scientific research, clinical diagnosis, pharmacogenomics research, and drug R&D, among others^7^.

It has been reported that approximately 48.5 million couples experience problems with infertility each year globally^8^. In recent years, with rapid economic growth and the acceleration of economic globalization, there have been increases in the proportion of women pursuing careers and the proportion of marriages that end in divorce. As such, childbearing has been put off until a later age, but reproductive capacity also declines with age. Taking all of these factors together, it is understandable that infertility rates continue to grow. Germline stem cells are germ cells at an early developmental stage, so their development is key to ensuring successful human reproduction. Germ stem cells are a group of cells that can transmit genetic information from parent to offspring and have the characteristics of both germ cells and stem cells^9,10^. Main examples of them include spermatogonial stem cells (SSCs) and female germline stem cells (FGSCs)^11^. Comprehensive transcriptome analyses can provide a solid foundation to understand the functions of lncRNAs and circRNAs in sex determination and the differentiation of male and female germline stem cells. Specifically, transcriptomic profiles of germline stem cells as reported in this paper should make a major contribution to the discovery of molecular markers, help to uncover the regulatory mechanisms of germline stem cells, and be useful as a reference for studies on the self-renewal and differentiation of such cells.

Although the expression profiles of lncRNAs and circRNAs in the mouse germline stem cells were provided in our previous study^12^, the current data descriptor provided more detailed descriptions of these unique lncRNA and circRNA transcriptomic datasets of the mouse germline stem cells, including both the methods used to collect the data and technical analyses supporting the quality of the measurements. Moreover, lncRNAs (especially, 9357 novel lncRNAs) were analyzed and annotated in a more precise manner (including interaction analysis of complementary lncRNA-mRNA^13^, up/down stream lncRNA of a gene^14–16^, pre-miRNA prediction^17^, and lncRNA family prediction^18^). More importantly, in view of the most important regulation mode of circRNA (circRNA as an endogenous competitive RNA (ceRNA) affecting the post-transcriptional regulation function of microRNAs), we made a thorough analysis of the interaction between circRNAs and microRNAs to provide the basis for subsequent functional experiments. In addition, tens of thousands of examples of single-nucleotide polymorphisms (SNPs), insertion–deletions (indels), alternative splicing (AS), and differential exon usage (DEU) were identified in these cells. This data descriptor will help advance data sharing and reuse to support reproducible research. The obtained data should also serve as an important reference for studying germ cell development and human reproduction, among others.

## Methods

### Animals

We purchased *Mvh-Cre* mice (FVB-Tg(*Ddx4-Cre*)1Dcas/J) and *mT/mG* mice (B6.129(Cg)-Gt*(Rosa)26Sor*^*tm4(ACTB-tdTomato,−EGFP*)Luo^/J) from Model Animal Research Center of Nanjing University. Mvh-Cre; mT/mG mice were produced using *Mvh-Cre* and *mT/mG* mice as mentioned previously^19^. Briefly, male *Mvh-Cre* mice were bred with female wild type females, while homozygous *mT/mG* mice were bred together. The *mT/mG* mice have two kinds of membrane-targeted fluorescent proteins (namely *tdTomato* and *EGFP)* at the *Rosa26* locus. Each side of the membrane-targeted tdTomato (mT) cassette has a *loxP* site, which shows red fluorescence in all the tissues. When *mT/mG* mice were hybridized with CRE-expressing mice, the tdTomato cassette was removed by CRE-mediated recombination and immediately began to express downstream membrane-targeted EGFP (mG) cassette in the CRE-expressing cells of the offspring. To obtain the *Mvh -Cre; mT/mG* mice, male *Mvh -Cre* mice (younger than 63 days old) were hybridized with female *mT/mG* mice. The *Mvh* -driven CRE was expressed in the germline lineage and led to a change in expression from tdTomato to EGFP in the germ cells of this strain. All procedures involving animals were approved by the Institutional Animal Care and Use Committee of Shanghai, and were conducted on the basis of the National Research Council Guide for Care and Use of Laboratory Animals.

### Mouse germline stem cell collection

**Fig. 1:**
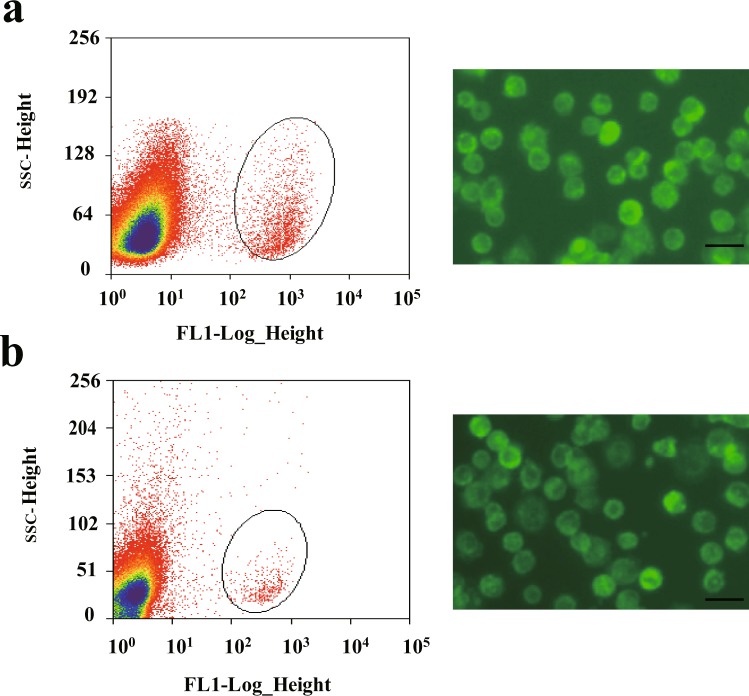
Isolation and purification of mouse SSCs and FGSCs. (**a**) Representative examples of SSCs purification with FACS and morphology of SSCs under fluorescence microscopy after purification. (**b**) Representative examples of FGSCs purification with FACS and morphology of FGSCs under fluorescence microscopy after purification. Scale bars: 20 μm.

To isolate and purify SSCs from mice, the testes of 50 male mice (Mvh-Cre; mT/mG mice, 6 days old) were collected and cut into small pieces. Then, SSCs were isolated by two-step enzymatic digestion, as mentioned previously^20^. After passing through a 13-µm nylon cell filter, the GFP-positive cells were sorted by flow cytometry (Fig. 1a). Lastly, GFP-positive cells were suspended in PBS and plated in 35 mm cell culture plate precoated with mouse laminin (4.4 µg/cm^2^). After incubated for 45 min at 37 °C, unbound cells were removed from bound cells by pipetting, and stored at −80 °C with proper amount of TRIzol reagent added. Fifty male mice were divided into 3 groups (16 or 17 mice for each group). All SSCs samples were collected in one day. To isolate and purify FGSCs from mice, the ovaries of 500 female mice (Mvh-Cre; mT/mG mice, 6 days old) were collected and cut into small pieces. Then, FGSCs were isolated by two-step enzymatic digestion, as mentioned previously^10,21^. After passing through a 13-µm nylon cell filter, the GFP-positive cells were sorted by flow cytometry (Fig. 1b). Lastly, GFP-positive cells were suspended in PBS and plated in 35 mm cell culture plate precoated with mouse laminin (4.4 µg/cm^2^). After incubated for 45 min at 37 °C, unbound cells were removed from bound cells by pipetting, and stored at −80 °C with proper amount of TRIzol reagent added. Five hundred female mice were divided into 3 groups (160–170 mice for each group). Each group of FGSCs samples were collected in 1 day.

### Characterization of mouse SSCs and FGSCs

**Fig. 2:**
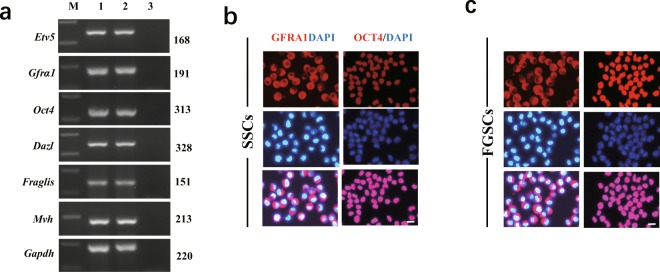
Characterization of mouse SSCs and FGSCs. (**a**) RT-RCR analysis of germline stem cell markers in SSCs and FGSCs. M, 250 bp DNA maker; lane 1, SSCs; lane 2, FGSCs; lane 3, no template control. (**b**) The SSCs was detected by immunofluorescence analysis with the antibodies against GFRA1 and OCT4. Scale bars: 10 μm. (**c**) The FGSCs was detected by immunofluorescence analysis with the antibodies against GFRA1 and OCT4. Scale bars: 10 μm.

To characterize these cells, we assessed marker gene expression of SSCs and FGSCs: *Gfrα1* (GDNF family receptor alpha 1), *Oct4* (also known as Pou5f1, POU domain class 5, transcription factor 1), *Etv5* (ets variant 5), *Dazl* (deleted in azoospermia-like), *Fragilis* (also termed Ifitm3, interferon induced transmembrane protein 3), and *Mvh* (also known as Ddx4, DEAD (Asp-Glu-Ala-Asp) box polypeptide 4). First, we determined the expression of these marker genes using reverse transcription PCR (RT-PCR). Total RNA was extracted from SSCs and FGSCs using Trizol reagent, according to the manufacturer’s protocol. Approximately 1000 ng RNA was used to synthesize cDNA (Complementary Deoxyribonucleic acid) using M-MLV reverse transcriptase in a 20 µl volume. PCR analysis was carried out with Taq DNA polymerase with primer sets specific for each gene^22^. The PCR conditions were 95 °C for 2 min, followed by 30 cycles comprising 95 °C for 30 s, 58 °C for 30 s, and 72 °C for 1 min, finally followed by 72 °C for 10 min, and storage at 4 °C. The glyceraldehyde −3- phosphate dehydrogenase (*GAPDH*) gene was amplified in each sample as a loading control. Samples were resolved through 1.5% agarose gels and run under the same conditions. DNA bands were detected using nucleic acid gel stain. RT-PCR results showed that the cells expressed *Gfrα, Oct4, Etv5, Dazl, Fragilis*, and *Mvh* (Fig. 2a). Then, we confirmed the expression of marker genes using immunofluorescence analysis. For this analysis, the cells cultured in 48-well plates were washed with 1× phosphate-buffered saline (PBS), fixed in 4% formaldehyde for 30 min at room temperature, and then washed three times with PBS for 5 min each. Then, the cells were incubated at 37 °C for 10 min in blocking buffer (PBS containing 10% goat serum). Next, the cells were incubated overnight at 4 °C with primary rabbit anti-GFRA1 antibody (1:100, ABclonal), or anti-OCT4 antibody (1:150, Santa Cruz). After washing three times with PBS, the cells were incubated at 37 °C for 30 min with a 1:150 diluted tetramethylrhodamine isothiocyanate (TRITC)-conjugated secondary antibody (goat anti-rabbit IgG; ProteinTech). The cells were incubated at 37 °C for 10 min with 500 ng/mL 4′,6-diamidino-2-phenylindole (DAPI; Sigma). Images were acquired using a Leica digital camera under a fluorescence microscope (DM2500, DMI3000B; Leica). Positive results were shown for GFRA1, and OCT4 proteins (Fig. 2b). All the characteristics clearly demonstrated the cells were actually pure population of SSCs and FGSCs.

### RNA extraction, cDNA library establishment, and Illumina sequencing

Total RNA was extracted from SSCs and FGSCs using Trizol reagent (Invitrogen, CA, USA), following the manufacturer’s protocol, and the RNA integrity number (RIN) was determined to evaluate RNA integrity using an Agilent Bioanalyzer 2100 (Agilent Technologies, Santa Clara, CA, USA). Ribosomal RNA was removed from total RNA to maximize the retention of all ncRNA. The obtained RNA was randomly cut into short fragments, and then this fragmented RNA was used as a template to synthesize the first strand of cDNA using random hexamers. The second strand was synthesized by adding buffer, dNTPs, RNase H, and DNA polymerase I. After purification using the QiaQuick PCR kit, end repair with EB buffer, single nucleotide A (adenine) addition, and connection with adapters, the second strand was finally degraded using uracil-N-glycosylase (UNG)^23^. Then, agarose gel electrophoresis was used to select fragment size. The suitable fragments were selected as templates for PCR amplification. Finally, a sequencing library was constructed and sequenced using the Illumina HiSeq 2000 platform (Table 1).

**Table 1:** Metadata of samples submitted to the NCBI Sequence Read Archive.

Source	Library strategy	Samples	Library layout	Platform	Instrument model	Biosample accession	Tissue
SSC	Strand-specific RNA-Seq	SSC-1	Paired	ILLUMINA	Illumina HiSeq 2000	SAMN05894751	Testis
SSC	Strand-specific RNA-Seq	SSC-2	Paired	ILLUMINA	Illumina HiSeq 2000	SAMN05894737	Testis
SSC	Strand-specific RNA-Seq	SSC-3	Paired	ILLUMINA	Illumina HiSeq 2000	SAMN05894735	Testis
FGSC	Strand-specific RNA-Seq	FGSC-1	Paired	ILLUMINA	Illumina HiSeq 2000	SAMN05894733	Ovary
FGSC	Strand-specific RNA-Seq	FGSC-2	Paired	ILLUMINA	Illumina HiSeq 2000	SAMN05894734	Ovary
FGSC	Strand-specific RNA-Seq	FGSC-3	Paired	ILLUMINA	Illumina HiSeq 2000	SAMN05894736	Ovary

### Filtering out “dirty” raw reads and alignment of reads to ribosomal RNA

To ensure the quality of data, raw data should be quality-controlled before information analysis, and data noise could be reduced by filtering. We here refer to reads containing an adapter, excessive “N,” or a large number of low-quality bases as “dirty” raw reads, which need to be removed before information analysis. The steps of filtering are as follows: 1) remove reads with adapters; 2) remove reads containing more than 10% “N”; and 3) remove low-quality reads (bases with Q < 10 constitute more than 50% of all reads). After filtering, 233,978,622 and 246,157,442 clean reads remained in the SSC and FGSC libraries, respectively, and were used for downstream bioinformatic analysis.

### lncRNA identification

**Fig. 3:**
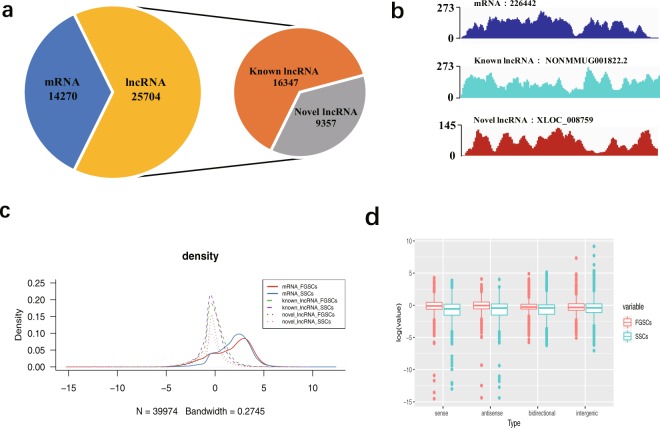
Identification and expression analysis of lncRNA candidates in germline stem cells. (**a**) A pie diagram showing the number of novel lncRNAs, known lncRNAs and mRNAs. (**b**) Examples of RNA-seq read density over random select mRNA, known lncRNA and novel lncRNA. (**c**) Represents the density of expression of known lncRNAs, novel lncRNAs and mRNAs in germline stem cells. N = 39974, Bandwidth = 0.2745. (**d**) Expression level of four types of lncRNAs.

We used the transcriptome data comparison software TopHat2^24^ to compare the filtered reads to the reference genome (UCSC mm10). Reads were assembled with Cufflinks^25^ after mapping to the genome. Considering the incomplete assembly of transcripts due to read coverage gaps, we performed reference annotation-based transcript (BRAT)^26^ assembly. The final assembled transcripts were compared with the reference gene, and the fragments that were roughly identical to the known transcripts were removed. After the assembly, we obtained the whole parsimonious set of transcripts; to detect the novel transcripts from the initial assembly, we compared the assembly transcripts to the reference annotation by utilizing Cuffcompare^25^. We utilized Cuffmege to merge several assemblies together; it automatically filtered a number of transfrags that were probably artifacts and produced a single annotation file for use in downstream differential analysis^27^. We evaluated and compared several software programs for lncRNA prediction, and chose Coding-Non-Coding Index (CNCI)^28^, Coding Potential Calculator (CPC)^29^, and iSeeRNA^30^, which performed well compared with the other software in both accuracy and efficiency. The findings that matched across all of the software were used to define novel lncRNA transcripts. For known lncRNA identification, we used the Gencode (GRCm38, M19) (https://www.gencodegenes.org/mouse/). The information on the annotation of lncRNAs in the Gencode database is currently more comprehensive than that in other databases, and the classification information of lncRNAs is more detailed. At the same time, we integrated 13 other databases to further verify the reliability of the lncRNAs: noncode (V4.0), RefSeq, UCSC (mm10), Ensemble, Hox_ncRNAs, Antisense_ncRNA_pipeline, fRNAdb, Affymetrix, TUCP, lncRNAdisease, eRNAs, ncRNA_imprint, and Hox_cluster_ncRNAs. The final potential lncRNAs were obtained by filtering the above basic properties and coding potential. Overall, 25704 expressed lncRNAs (16347 known and 9357 novel lncRNAs), 14270 expressed protein coding transcripts were identified and subjected to further analysis (Fig. 3a). The lncRNA identification data were deposited in figshare^22^. To display the distribution of lncRNA candidates along the chromosomes more intuitively, chromosome density distributions over the regions that were annotated as lncRNAs were analyzed statistically^22^. Moreover, we presented examples of RNA-seq read density over randomly selected mRNA, known lncRNA and novel lncRNA (Fig. 3b). Based on the fragments per kilobase of transcript per million mapped reads (FPKM) of each transcript, calculated by “Cufflinks” “abundance estimation mode” across SSCs and FGSCs, we compared the differences in expression among known lncRNAs, novel lncRNAs, and protein-coding genes. The average expression levels of lncRNAs were lower than those for protein coding genes while those of novel lncRNAs were similar to those of known lncRNAs (Fig. 3c). According to its location relative to nearby protein-coding genes, a lncRNA could be classified as sense overlap lncRNA, bidirectional lncRNA, antisense lncRNA, or intergenic lncRNA. When comparing the expression levels of the different classes of the lncRNAs, we found that the expression levels of lncRNAs of each type were similar (Fig. 3d).

**Fig. 4:**
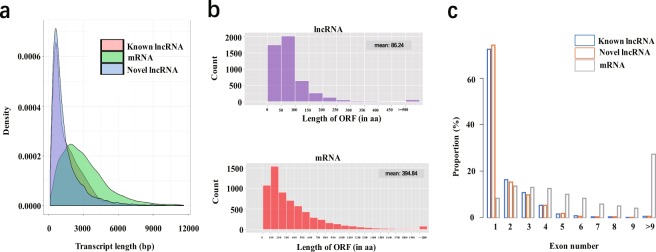
Sequence features and expression analysis of identified lncRNAs. (**a**) Length distribution of known and novel lncRNAs, and protein coding transcripts. (**b**) ORF distribution of lncRNAs and protein coding transcripts. (**c**) Exon number distribution of known and novel lncRNAs, and protein coding transcripts.

In order to analyze the structural charateristics of lncRNAs in the germline stem cells, we analyzed and compared the transcript length distribution, ORF length, and exon number between lncRNAs and mRNAs. Our analyses showed that transcript length distribution, ORF length, and exon number of lncRNAs were different from those of protein- coding transcripts. (Fig. 4a–c). The average transcript lengths of known and novel lncRNAs were much shorter than those of protein- coding transcripts. (Fig. 4a). Next, we compared the ORF length between the lncRNAs and the mRNAs. The principle of ORF analysis is based mainly on the six-frame translation of nucleic acids. Our results showed that the average ORF lengths of the lncRNAs and the mRNAs were 86.24 bp and 394.84 bp, respectively (Fig. 3b). This indicated that the ORF length of mRNAs were significantly longer than those of the lncRNAs. The main reason behind this is that lncRNAs do not encode proteins. Finally, the number of exons of lncRNAs and mRNAs was compared and analyzed, and the results showed that known and novel lncRNAs had fewer exons than mRNAs (Fig. 3c).

### lncRNA annotation and functional prediction

The annotation and functional prediction of lncRNAs were performed based on the mechanism of lncRNA function^17^: (1) Interaction analysis of complementary lncRNA–mRNA. To reveal the interaction between antisense lncRNA and RNA, we used RNAplex^31^, software for finding short interactions between two long strands of RNA, to predict complementary binding between antisense lncRNA and mRNA. The program includes the Vienna RNA package, and calculates the minimum free energy according to the thermodynamic structure to predict the best base pairing relationship. The results showed the best lncRNA–mRNA base pairing sites and the minimum free energy of antisense lncRNA and its corresponding mRNA. (2) For lncRNAs up/downstream of a gene, we annotated those classified as being located in an “unknown region” in the former analysis, if they were upstream or downstream of a gene. These lncRNAs could potentially overlap with *cis*-regulatory elements that are probably involved in transcriptional regulation. (3) Pre-miRNA prediction. We aligned lncRNAs to miRBase^32^ to detect potential pre-miRNAs, with those showing hit coverage higher than 90% being selected. Support vector machine (SVM)-based software miRPara^33^ was also used to predict probable miRNAs. It classified sequences from miRBase into animal, plant, and overall categories and used an SVM to train three models based on the physical properties of pre-miRNA and its miRNAs. (4) lncRNA family prediction. To better annotate lncRNA at the level of evolution, we used *INFERNAL* to classify all predicted lncRNAs according to their conserved sequence and secondary structure through multiple sequence alignment, secondary structure, and a covariance model^33^. The lncRNA annotation data were deposited in figshare^22^.

### CircRNA identification

**Fig. 5:**
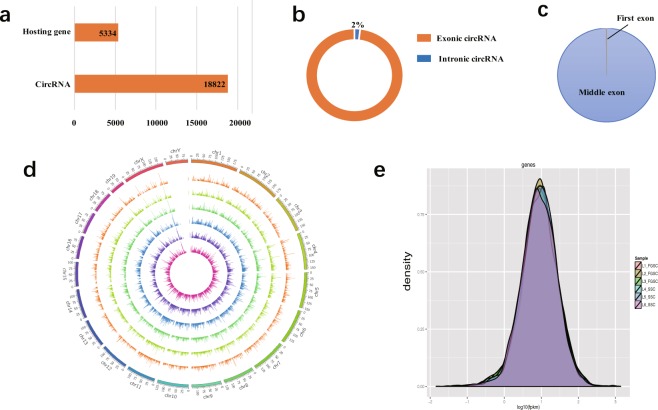
Sequence features of circRNAs. (**a**) Histogram showing the number of circRNAs and their hosting genes. (**b**) Ninety-eight percent of circRNAs were exonic circRNAs. (**c**) Distribution of the back-spliced exons in circRNAs. Nearly all (99.9%) back-spliced exons that contribute to circRNAs were located in the middle of their hosting genes, whereas 19 were in the first exon and none was in the last exon, as annotated. (**d**) Distribution of circRNA candidates in chromosomes. Each chromosome was mapped setting 25 MB as the basic unit. The expression of circRNAs in each segment was analyzed when the full array of circRNAs in different samples was visualized. SSC-1, SSC-2, SSC-3, FGSC-1, FGSC-2, and FGSC-3 were presented from the center of the circle outward, in this order. (**e**) Represents the density of expression of circRNAs in germline stem cells.

In accordance with the structural characteristics and splicing sequence characteristics of circRNAs, we used the following methods to identify them^34–36^: (1) Given the circular character of circRNAs, a database of junction reads was first established by using clean data that could not be compared with the mouse genome. With these junction reads as anchors, alternatively spliced transcripts were assembled by extending Cufflinks to both ends. (2) The assembled transcripts were divided into two sections centered on the junction reads and BLAST localization of the genome was performed; if the positions of the two sections in the mouse genome were reversed, the transcripts were considered as candidate circRNAs. (3) The candidate circRNAs were further filtered for protein-coding potential. The filtering parameters were as follows: Pysocsf (score < 100), CPC (score < 10), CNCI (score < 0), and Pofam (score < 0). Overall, 18822 expressed circRNAs derived from 5334 host genes were identified and subjected to further analysis (Fig. 5a). Among these 18822 circRNAs, 98% were exonic circRNAs (Fig. 5b). Moreover, the vast majority of exonic circRNAs identified were processed from exons in the middle of their hosting genes, with only a few including the first or the last exons (0.1% for the first exon, and none for the last exon) (Fig. 5c). The circRNA identification data were deposited in figshare^22^.

To display the distribution of circRNA candidates along the chromosomes more intuitively, we used Circos software (http://circos.ca/) to map the genomic locations of circRNAs screened as described above. We performed genome mapping according to different samples of circRNAs (Fig. 5d). We also analyzed the expression density of circRNAs in the germline stem cells. The results showed that the expression density of each sample conformed to the normal distribution and the average expression levels of circRNAs were lower than those of the protein- coding genes (Fig. 5e).

### CircRNA annotation and functional prediction

On the basis of the relationship between the locations of circRNAs in the genome and protein-coding genes, the screened circRNA candidates were annotated, which mainly involved categorical and functional annotations. The categorical annotations of circRNAs were mainly based on their positional relationships in the genome, which can be divided into five types: intronic circRNA, exonic circRNA, antisense circRNA, sense overlapping circRNA, and intergenic circRNA. The functional annotation of circRNAs was based on the mechanism by which the circRNAs form. This mainly involves the annotation of circRNA function according to the corresponding circRNA-hosting gene function, including gene function annotation, gene ontology (GO) annotation, and Kyoto Encyclopedia of Genes and Genomes (KEGG) annotation^35^. The circRNA annotation data were deposited in figshare^22^.

### Analysis of circRNA–miRNA interaction network

**Fig. 6:**
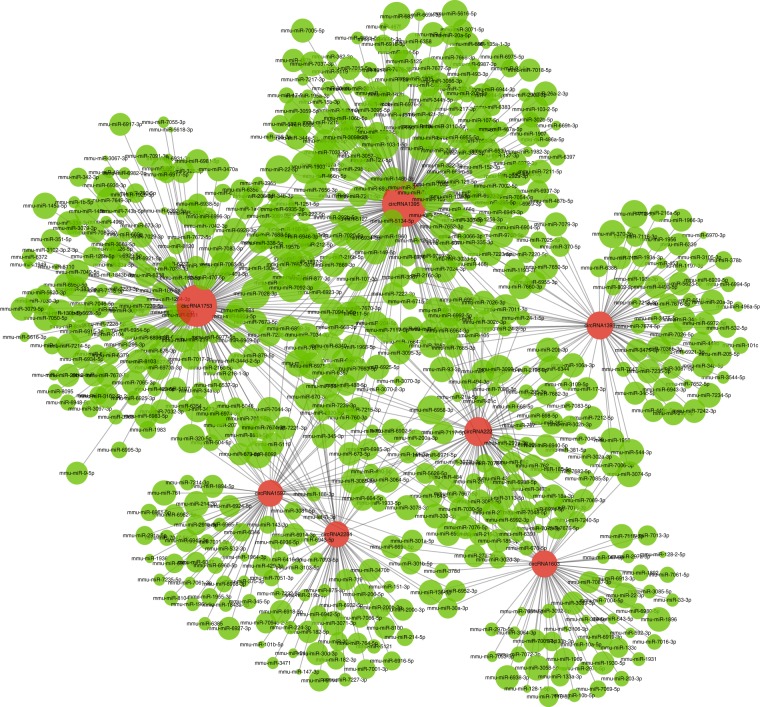
CircRNA–miRNA interaction network in germline stem cells. The panorama network consists of circRNAs (red circle) and miRNAs (green circle) in germline stem cells.

The analysis of interactions between circRNAs and miRNAs was mainly based on Targetscan 7.0 software (http://www.targetscan.org/) and miRanda software (http://www.microrna.org/microrna/home.do). The former software predicts the target of microRNAs based on the seed region, while the latter is mainly based on the size of the binding free energy between circRNA and miRNA. The smaller the binding free energy, the stronger the binding ability, and the screening threshold is max. energy ≤ −20. An entire circRNA–miRNA interaction network of germline stem cells was constructed^22^, part of which was delineated by Cytoscape (Fig. 6).

### Identification of SNPs, indels, AS, and DEU

**Fig. 7:**
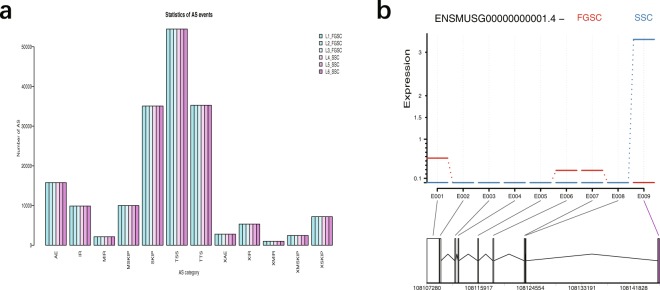
Analysis of alternative splicing and differential exon usage. (**a**) Classes and statistics of AS profile in germline stem cells. SKIP: exon skipping; MSKIP: cassette exons; IR: retention of single introns; MIR: retention of multiple introns; AE: alternative exon ends; TSS: alternative transcription start site; TTS: alternative transcription termination site. X in front of an abbreviation represents a blurred boundary. (**b**) An example of alternative exon usage analysis.

Based on data at the transcriptome level, the SNP loci in coding regions were analyzed. According to the results of the TopHat comparison between each sample and the reference genome, Samtools software^37^ was used to mpileup the possible SNP and indel information of each sample, and Annovar software^38^ was used to annotate it^22^. We selected Alternative Splicing Detector (ASD; available at http://www.novelbio.com/asd/ASD.html) as a tool to detect the cases with differential alternative splicing based on a bam file after mapping and using the mouse genome sequence as a reference, based on a *P*-value threshold of <0.05 (Fig. 7a). DEU analysis is currently used for alternative exon usage in alternative splicing^22^.In this study, DEXSeq software^39^ was used for DEU analysis (Fig. 7b). DEXSeq uses a generalized linear model to detect differentially expressed genes at the exon level^22^.

## Data Records

The identification, annotation data (circRNAs and lncRNAs) as well as SNPs, indels, AS, DEU and circRNA–miRNA interaction network were uploaded to figshare^22^. The original and normalized data associated with the samples analyzed in this study are deposited at Gene Expression Omnibus (GEO) datasets^40^.

## Technical Validation

### Quality control–RNA integrity

The RNA integrity number (RIN) of total RNA in germ stem cells was determined to evaluate RNA integrity using an Agilent Bioanalyzer 2100 (Agilent Technologies). The results showed that the RIN value was more than 7.0 for each sample. The values met the requirements for a noncoding RNA sequencing library.

### Quality validation and analyses

**Fig. 8:**
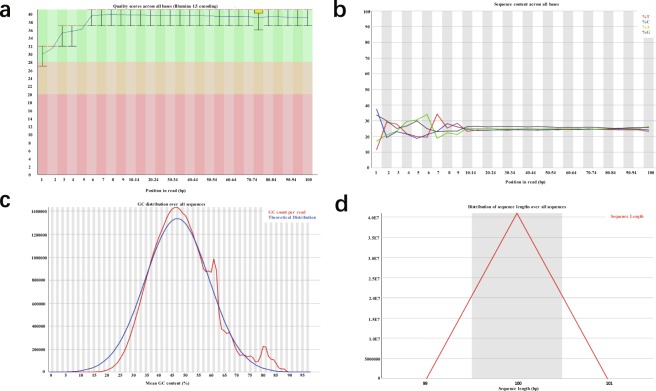
A representative example of quality control metrics of RNA sequence reads as indicated by FastQC (sample: FGSC-3). (**a**) Per base sequence quality. (**b**) Per base sequence content. (**c**) Per sequence GC content. (**d**) Sequence length distribution.

We applied FastQC v0.11.5 software to determine data quality and analyzed several variables reflecting this^41^. A representative summary plot is depicted (FGSC-3). Here, the per base sequence quality was high, with a median quality score above 30, suggesting high-quality sequences across all bases (Fig. 8a). We also created a figure of base composition and base quality for clean data. The results showed satisfactory base composition because the T curve is in accordance with the A curve; meanwhile, the C curve is in accordance with the G curve (Fig. 8b). The per sequence GC content was examined. The pattern of GC composition was similar to the theoretical distribution, indicating that the samples were free from contamination (Fig. 8c). In addition, the sequence length distribution also corresponded to the theoretical curve (Fig. 8d). All other FastQC files were shown to have similar quality metrics compared to sample FGSC-3.

The data provided in these experimental datasets are the first reported genome-wide lncRNA and circRNA transcriptome resources for male and female mouse germline stem cells, which include SNPs, indels, AS, and DEU analyzed by Illumina high-throughput sequencing technology. These findings are useful for the identification of lncRNAs and circRNAs related to the self-renewal and sex-specific properties required for differentiation into gametes, as well as the development of polymorphic genetic markers in germline stem cells and other related research.

## ISA-Tab metadata file

Download metadata file
